# Role of Maximum Standardized Uptake Volume in Predicting Tumor Spread Through Air Spaces in Stage IA Lung Cancers Smaller than 2 cm

**DOI:** 10.3390/cancers18091480

**Published:** 2026-05-05

**Authors:** Massimiliano Bassi, Beatrice Zacchini, Rita Vaz Sousa, Angelina Pernazza, Paolo Graziano, Silvia De Maria, Silvia Albano, Camilla Poggi, Marco Anile, Tiziano De Giacomo, Federico Venuta, Daniele Diso

**Affiliations:** 1 Thoracic Surgery Unit, Department of General Surgery, Surgical Specialties and Anestesiology, Policlinico Umberto I, Sapienza University of Rome, 00161 Rome, Italy; beatrice.zacchini@uniroma1.it (B.Z.); rita.ferreira@uniroma1.it (R.V.S.); silvia.demaria@uniroma1.it (S.D.M.); silvia.albano@uniroma1.it (S.A.); camilla.poggi@uniroma1.it (C.P.); marco.anile@uniroma1.it (M.A.); tiziano.degiacomo@uniroma1.it (T.D.G.); federico.venuta@uniroma1.it (F.V.); daniele.diso@uniroma1.it (D.D.); 2 Department of Radiological, Oncological and Pathological Sciences, Sapienza University Rome, 00161 Rome, Italy; angelina.pernazza@uniroma1.it (A.P.); paolo.graziano@uniroma1.it (P.G.)

**Keywords:** lung cancer, STAS, PET, maximum standardized uptake volume, lobectomy, segmentectomy

## Abstract

Objective: Recent guidelines suggest sublobar resection as a viable option for peripheral lung cancer smaller than 2 cm. However, the presence of Spread through Air Spaces (STAS) is a well-known poor prognostic factor in early-stage lung cancer treated with sublobar resections. The aim of this study is to analyze the potential correlation between STAS and maximum standardized uptake volume (SUVmax) in this subset of patients to help define the optimal resection. Methods: A retrospective monocentric study was performed including patients diagnosed with stage IA lung cancer undergoing surgical resection. Patients were divided into two groups according to the presence/absence of STAS. As further investigation, we also separately analyzed the subgroup of patients with a peripheral nodule smaller than 2 cm. A *p*-value ≤ 0.05 was considered statistically significant. Results: The study cohort consists of 121 patients, 76 (62.8%) male, with a mean age of 74.2 ± 8.8 years. STAS was observed in 67 (55.4%) cases. The STAS-positive group showed a higher SUVmax value compared to the STAS-negative one (mean 5.5 ± 4.4 vs. 3.9 ± 3.0; *p*-value 0.007) with an AUC of 0.65 and an optimum SUVmax cut-off value of 3.0. Moreover, patients with SUVmax ≥ 3 showed a 77% increase in risk of having STAS (RR = 1.77; 95%CI = 1.21–2.59, *p*-value 0.014). This association was confirmed also in the subgroup of patients with nodules ≤ 2 cm (mean SUVmax 5.3 ± 4.6 vs. 3.6 ± 2.7; *p* value = 0.014). Conclusions: In our study, an SUVmax ≥ 3 in preoperative 18-FDG-PET is associated with the presence of STAS in stage IA lung cancer and peripheral cancer smaller than 2 cm. These findings, integrated with other clinical and radiological data, may guide surgeons to optimize the surgical strategy.

## Simple Summary

This study explores the role of 18F-fluorodeoxyglucose Positron Emission Tomography (18F-FDG-PET) as a preoperative predictor of Spread through Air Spaces (STAS) in early-stage non-small cell lung cancer (NSCLC), with the aim of improving surgical decision-making. STAS has emerged as an adverse prognostic factor, particularly after sublobar resections. This is particularly relevant for peripheral tumors ≤ 2 cm, where recent evidence supports sublobar resection as a feasible alternative to lobectomy. However, most available data derive from ground-glass opacity lesions, and evidence regarding PET-positive nodules remains limited. In this context, our study specifically investigates the predictive role of 18F-FDG-PET in stage IA NSCLC, focusing on tumors ≤ 2 cm. We identified an SUVmax ≥ 3 as significantly associated with the presence of STAS. These data suggest that, in patients with small peripheral tumors but elevated SUVmax, the presence of STAS should be considered to optimize the surgical strategy.

## 1. Introduction

Early diagnosis is the key factor for complete resection and potential cure in the case of lung cancer, a worldwide cause of tumor-related death [1]. In resectable early-stage non-small cell lung cancer (NSCLC), lobectomy still represents the gold standard [2,3]. However, in the case of small, peripheral and subsolid nodules, sublobar resections, particularly segmentectomy, have shown comparable outcomes and are now considered a viable and effective therapeutic alternative [2,3,4,5].

Spread through Air Spaces (STAS) has recently gained clinical attention due to its association with a worse prognosis and an increased risk of local recurrence, particularly in patients undergoing limited resections [6,7]. Intraoperative diagnosis of STAS could make the difference, but it is often unreliable; thus, it is crucial to develop tools for an effective preoperative assessment [8,9,10].

In this context, 18F-fluorodeoxyglucose Positron Emission Tomography (18F-FDG PET) has proven to be a promising noninvasive method to provide both morphologic and metabolic information. Recent studies showed the predictive value of variables such as SUVmax and consolidation-to-tumor ratio to identify STAS-positive tumors [11,12,13,14,15,16]. In addition, the integration of PET variables with traditional computed tomography (CT) imaging allowed the development of promising predictive models for risk stratification. However, studies specifically focusing on stage IA NSCLC remain limited.

Our study aims to investigate the association between 18F-FDG PET and STAS in patients diagnosed with stage IA lung cancer. As a further investigation, we analyzed the subgroup of patients with a peripheral cancer smaller than 2 cm. In those patients, guidelines indicate that both lobectomy and segmentectomy/wedge resection are considered viable options; having the possibility to know STAS preoperatively may change the surgical strategy.

## 2. Materials and Methods

We retrospectively reviewed a group of patients with lung cancer undergoing surgery at our institution between January 2017 and December 2023, according to the following inclusion criteria: preoperative 18-FDG PET scan within 3 months before surgery; pathological stage IA NSCLC according to the 9th TNM classification of the International Association for the Study of Lung Cancer; and assessment of STAS in the surgical specimen.

Patients with synchronous lung lesions, lung cancer recurrence or large cell neuroendocrine carcinoma and other rare lung tumors were not included.

STAS was defined in histopathological specimens according to the International Association for the Study of Lung Cancer as the presence of tumor cells within air spaces in the lung parenchyma beyond the edge of the main tumor. Histological evaluation was performed on formalin-fixed, paraffin-embedded, hematoxylin and eosin-stained sections. STAS assessment was performed by two experienced thoracic pathologists blinded to imaging data.

We anonymously collected the following clinical and radiological data: demographics (age, sex), medical history (smoking habits, previous diseases, comorbidities), imaging data (maximum diameter, aspect of the nodule, SUVmax), pathological data (tumor histotype and subtypes, tumor size, grading, presence or absence of STAS), and surgical procedure (lobectomy, segmentectomy or wedge resection; surgical approach).

The statistical analysis was performed using IBM SPSS Statistics v.27 (IBM Corp., Armonk, NY, USA). Categorical data are presented as numbers and percentages; continuous data as mean ± standard deviation (SD). The Shapiro–Wilk test was used to ensure the normal distribution of continuous data. The chi-square test was used to compare categorical data while Student’s *t*-test and the Mann–Whitney U test were applied to compare normally distributed and non-normally distributed continuous data, respectively. The Youden Index was utilized to determine the optimal cut-off value in the Receiver Operating Characteristic (ROC) curve. Risk ratios (RRs) were computed by comparing event probabilities across groups using contingency tables. Odds ratios (ORs) with 95% confidence intervals (CIs) were obtained through univariable logistic regression. Multivariable analysis was performed using logistic regression models to identify independent predictors of STAS. Due to collinearity between SUVmax as a continuous variable and the dichotomized variable SUVmax ≥ 3, two separate multivariable models were developed. The following covariates were included based on clinical relevance and univariable analysis results: tumor size, grading, radiological appearance, and SUVmax (continuous and dichotomized). Results were reported as odds ratios (ORs) with 95% CIs. A *p*-value ≤ 0.05 was considered statistically significant.

This study was approved by the Ethical Committee of Policlinico Umberto I (Protocol Number 1020/2024). Considering the retrospective nature of the study, the requirement of a specific informed consent was waived.

## 3. Results

**Figure 1:**
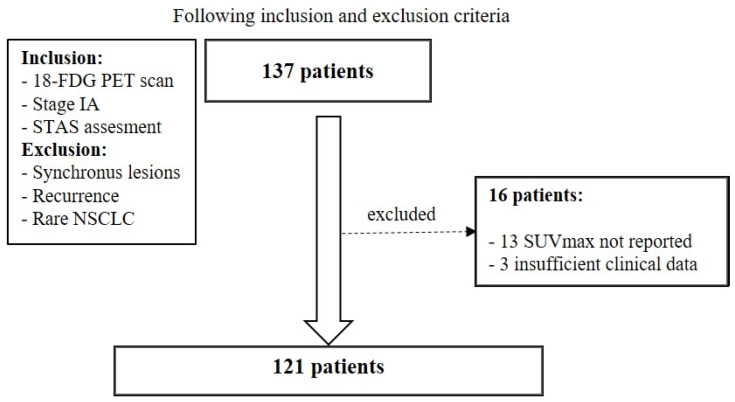
Flow diagram of study enrollment.

A hundred thirty-seven patients were initially included in the study. Among them, 13 patients were excluded because SUVmax was not explicitly indicated, and 3 because clinical data were incomplete (Figure 1).

Therefore, the final cohort resulted in 121 patients, 76 (62.8%) male and 45 (37.2%) female, with a mean age of 74.2 ± 8.8 years. Among them, 62 (51.2%) were ex-smokers, 40 (33.0%) were current smokers, and 19 (15.7%) were never smokers. Hypertension (59.5%), dyslipidemia (52.0%), COPD (43.8%) and diabetes (29.8%) were the most frequently observed comorbidities, resulting in an ASA I score in 7.4%, an ASA II score in 43.8% and an ASA III score in 48.8% of the patients. All of them underwent surgical resection: 52 (42.9%) received lobectomy, four (3.3%) segmentectomy, and 65 (53.7%) wedge resection. The tumor was in the right upper lobe in 37 (30.5%) patients, in the middle lobe in four (3.3%), in the right lower lobe in 26 (21.4%), in the left upper lobe in 34 (28%), and in the left lower lobe in 20 (16.5%). All patients underwent a lobe-specific mediastinal lymph node dissection as the institutional standard approach. CT scan analysis showed a solid lesion in 59 (48.8%) cases, a mixed nodule in 44 (36.4%), and pure ground glass opacity in 18 (14.9%) cases. Histology showed adenocarcinoma in 100 (82.6%) patients, squamous cell carcinoma in 20 (16.5%) and mucoepidermoid carcinoma in one (0.8%). Adenocarcinomas showed an acinar predominant pattern in 58 (58.0%), a solid predominant pattern in 17 (17.0%), a lepidic predominant pattern in 10 (10.0%), a papillary predominant pattern in eight (8.0%) and others in seven (7.0%). Three patients with a lepidic predominant pattern received a diagnosis of minimally invasive adenocarcinoma (MIA). Sixty-three patients showed a moderately differentiated cancer (G2, 52.1%), 50 were poorly differentiated (G3, 41.3%), and four had a well-differentiated tumor (G1, 3.3%). The mean tumor size was 16.8 ± 5.8 mm (range 4–29 mm). No perioperative mortality was observed. The mean postoperative hospital stay was 5.8 ± 3.9 days. Postoperative complications were observed in 21 (17.3%) patients, most commonly cardiac arrhythmia (6, 5.0%) and prolonged air leaks (3, 2.5%).

All the patients underwent preoperative 18-FDG PET. Overall, the mean SUVmax was 4.8 ± 3.9 (range 1–27.6). STAS was found in the surgical specimen in 67 (55.4%) cases, while 54 (44.6%) patients were STAS negative. In our cohort, STAS was not statistically correlated with sex, age, smoking history and histological subtype as shown in Table 1. However, STAS was significantly associated with tumor grading (*p*-value = 0.005), acinar and solid adenocarcinoma pattern (*p*-value 0.031) and the presence of solid lesions at CT scan (*p*-value 0.041) (Table 1).

### 3.1. Correlation Between Stas and Suvmax Values

Patients with STAS showed significantly higher SUVmax values compared to those without STAS (5.5 ± 4.4 vs. 3.9 ± 3.0; *p* = 0.007).

The ROC curve analysis showed an AUC (area under the curve) of 0.65 with an optimum SUVmax cut-off value measured through the Youden Index of 3.0.

Therefore, patients were dichotomized into two groups according to whether SUVmax was higher or lower than 3. In the SUVmax ≥ 3 group, 47 patients (68.1%) were STAS positive, while 22 (31.9%) were STAS negative. Conversely, among patients with an SUVmax < 3, 20 (38.5%) were STAS positive and 32 (61.5%) were negative. This difference resulted in a statistically significant result, with a *p*-value of 0.001 after chi-squared analysis. Moreover, the SUVmax ≥ 3 group showed an RR of 1.77 (95% CI, 1.21–2.59), indicating a 77% increase in risk of having STAS compared to SUVmax < 3. Univariate logistic regression confirmed this association, yielding an OR of 3.42 (95% CI, 1.61–7.27).

After multivariable analysis including tumor size, grading, and radiological appearance, both SUVmax (as a continuous variable) and SUVmax ≥ 3 were independently associated with STAS. In particular, SUVmax showed an OR of 1.16 (95% CI 1.03–1.31; *p* = 0.015), while SUVmax ≥ 3 was associated with a more than two-fold increased risk of STAS (OR 2.94, 95% CI 1.32–6.54; *p* = 0.008). Tumor grading also remained independently associated with STAS, whereas tumor size and radiological appearance were not significant predictors (Table 2).

### 3.2. Analysis of the Subgroup of Patients with T ≤ 2 cm

As a further investigation, we performed the analysis in the subgroup of patients with a peripheral nodule smaller than 2 cm in diameter. This cohort consisted of 94 patients (76 (80.6%) male; mean age 73.8 ± 8.62 years) with a mean tumor diameter of 14.4 ± 3.9 mm. Thirty-seven (39.4%) underwent lobectomy and 57 (60.6%) received segmentectomy/wedge resection. Histological examination showed adenocarcinoma in 78 patients (83.0%) and squamous cell carcinoma in 16 patients (17.0%). The most frequent prevalent pattern in adenocarcinoma was acinar in 44 patients (56.4%), solid in 13 (16.7%) and lepidic in 8 (10.3%). STAS was present in 51 (54.3%) patients. A statistically significant association between the presence of STAS and SUVmax was confirmed also in this subgroup of patients: the STAS-positive and -negative groups showed a mean SUVmax of 5.3 ± 4.6 and 3.6 ± 2.7 respectively (*p* value = 0.014). The SUVmax ≥ 3 group showed a significantly higher STAS (65.4% vs. 40.5%; *p* value = 0.016) with a 67% increase in risk of having STAS than the SUVmax < 3 group (RR 1.61, 95% CI 1.06–2.44). Multivariable logistic regression analysis confirmed that both SUVmax (OR 1.14, 95% CI 1.01–1.30; *p* = 0.041) and SUVmax ≥ 3 (OR 2.71, 95% CI 1.13–6.47; *p* = 0.025) were independent predictors of STAS also in this subgroup of patients.

## 4. Discussion

In this study, we assessed the potential correlation between 18F-FDG PET parameters and STAS in stage IA NSCLC, particularly those smaller than 2 cm.

Previous studies showed a possible relationship between STAS and metabolic 18F-FDG PET parameters in lung cancer [10,11,12,13,14], but just a few reports focused on stage I NSCLC. Nishimori et al. [15] proved that SUVmax is the most reliable metabolic parameter to predict STAS in clinical stage I lung adenocarcinoma with a sensitivity of 89.5%, a specificity of 52.8% and an AUC of 0.74 using an SUVmax cut-off of 2.48.

In a recent study, Wang Y and colleagues [17] showed the predictive value of SUVmax and consolidation-tumor ratio in determining the presence of STAS in stage IA NSCLC, creating a nomogram with 78.1% accuracy in their validation test.

Suh JW et al. [16] proposed a stepwise flowchart using the SUV tumor/liver ratio at 18F-FDG PET to predict STAS negativity in NSCLC with T ≤ 2 cm achieving good results. However, their cohort also included pathological T2a, N1 and N2 patients, thus mixing stages IB, IIB and IIIA in the analysis.

While previous studies have demonstrated an association between PET-derived features and STAS, to our knowledge this is the first study specifically assessing this correlation in stage IA lung cancer smaller than 2 cm. This specific cohort of patients is clinically relevant, as sublobar resection is increasingly considered a valid alternative to lobectomy.

Our analysis showed how a preoperative SUVmax ≥ 3 is strongly correlated with the STAS in the pathological specimen. This information could be crucial in the case of small peripheral NSCLC to better personalize the surgical treatment considering that many studies showed that STAS is associated with worse prognosis and increased local recurrence after sublobar resection [5,6]. Importantly, the association between SUVmax and STAS persisted after adjustment for tumor size, grading, and radiological appearance, suggesting that metabolic activity reflects tumor aggressiveness beyond conventional clinicopathological features.

However, the discriminatory performance of SUVmax alone was limited, reaching an AUC of 0.65. Moreover, even if consistent with the literature, the SUVmax ≥ 3 cut-off is data-driven and needs validation. These findings indicate that SUVmax alone provides limited discriminatory value and may be better interpreted within a multimodal framework [18,19,20]. Advanced approaches such as radiomics and artificial intelligence-based tools may improve STAS preoperative prediction by integrating metabolic, radiological, and clinical data [13,14,21]. However, these tools are not yet widely implemented in clinical practice due to limited external validation, lack of standardization, and concerns regarding generalizability [9,22].

This study presents some limitations. First, it is a retrospective single-center study, potentially leading to a selection bias in patient selection, surgical strategy (lobectomy vs. segmentectomy vs. wedge) and STAS assessment. Interestingly, our cohort shows a relatively high prevalence of STAS in stage IA NSCLC. This finding is likely related to the PET-based selection of patients and the high predominance of solid nodules. In addition, PET/CT examinations were performed across different centers according to routine clinical practice, resulting in heterogeneity in acquisition protocols and representing a potential source of bias. Second, this study focused only on the correlation between SUVmax and STAS, not considering other 18-FDG PET parameters. We chose SUVmax because it is the most common metabolic feature measured at 18-FDG PET/CT and other parameters are not routinely reported. In addition, we did not perform a survival analysis because of incomplete follow-up data and thus discussion on surgical strategy must be interpreted cautiously and in the context of existing literature. Finally, the cohort number is relatively small and larger multi-centric studies are required to confirm these results.

## 5. Conclusions

In conclusion, an SUVmax ≥ 3 on preoperative 18-FDG PET was associated with the presence of STAS in stage IA NSCLC and in peripheral lung cancers smaller than 2 cm. These findings might improve preoperative risk stratification, particularly in early-stage lung cancer patients who may be candidates for sublobar resection. Integration of SUVmax data within the clinical and radiological assessment could offer an additional guide to plan the most appropriate surgical approach.

## Figures and Tables

**Table 1:** Correlation analysis between STAS-positive and STAS-negative groups.

Characteristics	STAS Positive	STAS Negative	*p*-Value
Patients (*n*)	67 (55.4%)	54 (44.6%)	
Age (years)	73.0 ± 9.9	75.8 ± 7.1	0.087
Sex Male Female	40 (33.1%) 27 (22.3%)	36 (29.8%) 18 (14.9%)	0.431
Smoking status Former Current Never	36 (29.8%) 22 (18.2%) 9 (7.4%)	26 (21.4%) 18 (14.9%) 10 (8.3%)	0.677
Tumor diameter (mm)	17.1 ± 5.6	16.4 ± 6.0	0.446
Radiological appearance Solid/Mixed Ground glass	61 (50.4%) 6 (5.0%)	42 (34.7%) 12 (9.9%)	**0.041**
SUVmax	5.5 ± 4.4	3.9 ± 3.0	**0.007**
SUVmax > 3 Yes No	47 (68.1%) 20 (38.5%)	22 (31.9%) 32 (61.5%)	**0.001**
Histology Adenocarcinoma Squamous cell	58 (47.9%) 9 (7.4%)	41 (33.9%) 13 (10.7%)	0.131
Grading G1 G2 G3	0 (0%) 32 (26.4%) 35 (28.9%)	4 (3.3%) 31 (25.6%) 16 (13.2%)	**0.005**

STAS = Spread through Air Spaces; SUVmax = maximum standardized uptake volume. *p*-values ≤ 0.05 (in bold) were considered statistically significant.

**Table 2:** Multivariable logistic regression analysis for predictors of STAS.

Variable	OR (95% CI)	*p*-Value
Model 1 (SUVmax continuous)		
SUVmax	1.16 (1.03–1.31)	**0.015**
Tumor size (mm)	1.04 (0.96–1.13)	0.318
Grading (G1–G3)	2.48 (1.20–5.10)	**0.014**
Solid vs. GGO	2.21 (0.78–6.22)	0.134
Model 2 (SUVmax ≥ 3)		
SUVmax ≥ 3 (yes/no)	2.94 (1.32–6.54)	**0.008**
Tumor size (mm)	1.03 (0.95–1.12)	0.441
Grading (G1–G3)	2.26 (1.13–4.93)	**0.022**
Solid vs. GGO	2.39 (0.84–6.78)	0.103

Due to collinearity, SUVmax (continuous) and SUVmax ≥ 3 were included in separate models. STAS = Spread through Air Spaces; OR = odds ratio; CI = confidence interval; GGO = ground-glass opacity. *p*-values ≤ 0.05 (in bold) were considered statistically significant.

## Data Availability

The data underlying this article will be shared on reasonable request to the corresponding author.

## Abbreviations

The following abbreviations are used in this manuscript:

NSCLC	non-small cell lung cancer
STAS	Spread through Air Spaces
18-FDG PET	18F-fluorodeoxyglucose Positron Emission Tomography
CT	computed tomography
SD	standard deviation
ROC	Receiver Operating Characteristic
RR	Risk ratio
OR	Odds ratio
CI	confidence intervals
MIA	minimally invasive adenocarcinoma
AUC	area under the curve
